# Survey on Anxiety and Post-Traumatic Stress Disorder in Intensive Care Personnel during the COVID-19 Pandemic in a Medically Under-Resourced Country

**DOI:** 10.3390/healthcare10071160

**Published:** 2022-06-22

**Authors:** Alexandra Elena Lazar, Janos Szederjesi, Oana Coman, Andrea Elekes, Mariana Suciaghi, Bianca Liana Grigorescu

**Affiliations:** 1 Department of Anesthesiology and Intensive Care, George Emil Palade University of Medicine, Pharmacology, Science and Technology, 540139 Târgu Mureș, Mures County, Romania; alexandra.lazar@umfst.ro; 2 Department of Simulation Applied in Medicine, George Emil Palade University of Medicine, Pharmacology, Science and Technology, 540139 Târgu Mureș, Mures County, Romania; oana.coman@umfst.ro; 3 Department of Anesthesiology and Intensive Care, Emergency Clinical County Hospital, 540136 Târgu Mureș, Mures County, Romania; e.andrea92@yahoo.com; 4 Department of Anesthesiology and Intensive Care, Psychology, Emergency Clinical County Hospital, 540136 Târgu Mureș, Mures County, Romania; mari_suciaghi@yahoo.com; 5 Department of Pathophysiology, George Emil Palade University of Medicine, Pharmacology, Science and Technology, 540142 Târgu Mureș, Mures County, Romania; bianca.grigorescu@umfst.ro

**Keywords:** intensive care, anxiety, post-traumatic stress disorder, COVID-19, medical personnel

## Abstract

The COVID-19 pandemic has increased the need for intensive care personnel. Romania has a low number of physicians per inhabitant. The stress of pandemics upon an already weak medical health system triggered some psychological effects upon burnt-out personnel. The main objective is to provide an insight into the psychological status of Romanian ICU personnel by evaluating their level of anxiety. The secondary objectives aim to identify the level of post-traumatic stress disorder and anxiety in different groups and to identify the personnel most affected psychologically. This study enrolled adult responders from the ICU of Târgu Mureș Emergency Clinical County Hospital, Romania, participating voluntarily. The evaluation tests were the State-Trait Anxiety Inventory and Post-Traumatic Stress Test. Out of the 126 eligible participants, 87 adult employees were enrolled—with a 69% response rate. The study comprised three groups: doctors, nurses, and auxiliary personnel. All three groups scored for moderate anxiety symptoms. COVID-19-related anxiety was strongly correlated with age and number of working years in all groups. Increased PTSD scores were observed in doctors and nurses. All ICU personnel who dealt with COVID-19 patients presented with moderate anxiety and post-traumatic stress disorder symptoms. The years of ICU experience had a positive impact on anxiety symptoms.

## 1. Introduction

Worldwide, the COVID 19 pandemic has increased the need for intensive care personnel. Romania, an eastern European country, has a low number of physicians per number of inhabitants. The numbers provided by statista.com [1] in 2020 show that there were roughly 66 thousand physicians in Romania and 152,686 people working in the Romanian medical system as auxiliary medical staff. Matching these statistics, we see that in 2020 there were only three physicians per 1000 inhabitants in Romania [1]. Compared to other European countries, such as Sweden (5.39/1000 inhabitants), Austria (5.14/1000 inhabitants), Greece (4.52/1000 inhabitants), Norway (4.49/1000 inhabitants), and Denmark (4.45/1000 inhabitants), it can be understood that the medical personnel from Romania bear a supplementary burden from understaffing [2,3]. It is undoubted that this workload is an important source of stress, anxiety, and burnout. On top of this, the pandemic has produced serious damage to healthcare providers. By May 2021, the number of infected healthcare personnel in the Romanian healthcare system reached 33,968, according to statista.com [1].

Intensive care units have been frontline battlefields during the COVID-19 pandemic, where they have been under constant pressure since the beginning [3]. The stress of the pandemic upon an already weak medical health system triggered some psychological effects upon already burnt-out personnel. Because of this, symptoms of anxiety or post-traumatic stress disorder could be observed among medical and non-medical personnel [4,5]. ICU stress levels rose during the pandemic and the healthcare workers were exposed to depression, stigmatization, fear, or exhaustion. This distress was first the result of the unknown disease they were fighting and second due to the lack of a known cure at the beginning of the pandemic [3,4,5,6,7,8].

Although these observations can be easily inferred, little is known about the psychological effects of COVID-19 on ICU healthcare personnel. This aspect is important because the mental health of doctors, nurses, and auxiliary personnel could make a real difference when it comes to caring for patients. Burnt-out doctors and nurses can have problems fulfilling their tasks; they may experience symptoms of futility regarding their work and their purpose on the job, aspects which are ultimately detrimental to the patients [9,10].

The stress experienced by healthcare personnel working in COVID ICUs is high and has a great impact on caregivers [9,10].

This situation appears because of the dramatic situation of the patients combined with the desperation of the families, who are not able to be with the loved ones in their hardest moments. The enormous everyday workload in association with the psychological impact of COVID-19 patients has a negative influence—depression, lack of motivation, and reduced task performance—on ICU doctors, nurses, and other personnel.

This paper has the main objective to provide an insight into the psychological status of Romanian ICU personnel by evaluating their level of anxiety—basic and event-related.

As a secondary objective, it aims to identify the existence and the level of post-traumatic stress disorder and anxiety levels.

Another secondary objective consists in comparing the level of stress and anxiety among different groups of medical personnel to identify where the pandemics had a greater psychological impact.

## 2. Materials and Methods

This prospective non-randomized, survey-type study enrolled 87 responders from the ICU of the Târgu Mureș Emergency Clinical County Hospital, Romania. The study was carried out in March 2021. The Ethics Committee was waived. The responders were all adults and participated voluntarily in the study.

The inclusion criteria were:

- ICU employee.
- Willingness to participate.

The exclusion criteria were:

- Other hospital employees—not ICU.
- Refusal to participate.
- History of psychiatric disorders.

The data were gathered by handing out questionnaires that were then anonymously filled by the participants (Supplementary Material Questionnaire S1). We analyzed the following data: age, number of working years, anxiety test results, Post-Traumatic Stress Test results, and time spent in COVID units. The time spent in the COVID unit represents the number of days worked in the COVID unit, reported by the employees and confirmed by their work charts.

The State-Trait Anxiety Inventory (STAI) is a commonly used measure of trait and state anxiety developed by Spielberger et al. [11].

The questionnaires comprised three distinct sections:

- The Post-Traumatic Stress Test (TPST)—comprises 13 items that were evaluated from 0 (low) to 4 (high). The highest possible score is 52 and the lowest is 0.

Interpretation of TPST points:

- 0–12—no posttraumatic stress symptoms.
- 13–26—few symptoms.
- 27–40—moderate symptomatology.
- 41–52—significant post-traumatic stress symptoms.

- State-Trait Anxiety Inventory (STAI) is a psychological inventory that measures two types of anxiety: STAI-1—state anxiety, or anxiety about an event, and STAI-2—state anxiety, or anxiety as a feature. Each of these scales contains 20 items, scored with points from 1 (rarely) to 4 (always). The minimum score is 20 and the maximum is 80 points.

Interpretation of STAI points:

- 20–35—minimum anxiety.
- 36–50—moderate anxiety.
- 51–65—increased anxiety.
- 66–80—high anxiety.

Data from the questionnaires were introduced into Excel sheets and statistical analysis was performed with Graph Pad Prism 8 (GraphPad Software Inc., San Diego, CA, USA). The statistic tests used for data processing were the Kruskal–Wallis test for non-parametric, non-paired data and Spearman correlations. The statistical significance level was set at *p* < 0.05 and an interval of confidence (CI) of 95%. The results from the anxiety tests were also evaluated by a certified psychologist who works daily in our ICU clinic.

## 3. Results

The calculated sample size was 96 responders from the eligible population of 126. We obtained 87 survey responses, which extended our margin of error for the sample size to 5.87 instead of the usual set of 5%.

**Figure 1:**
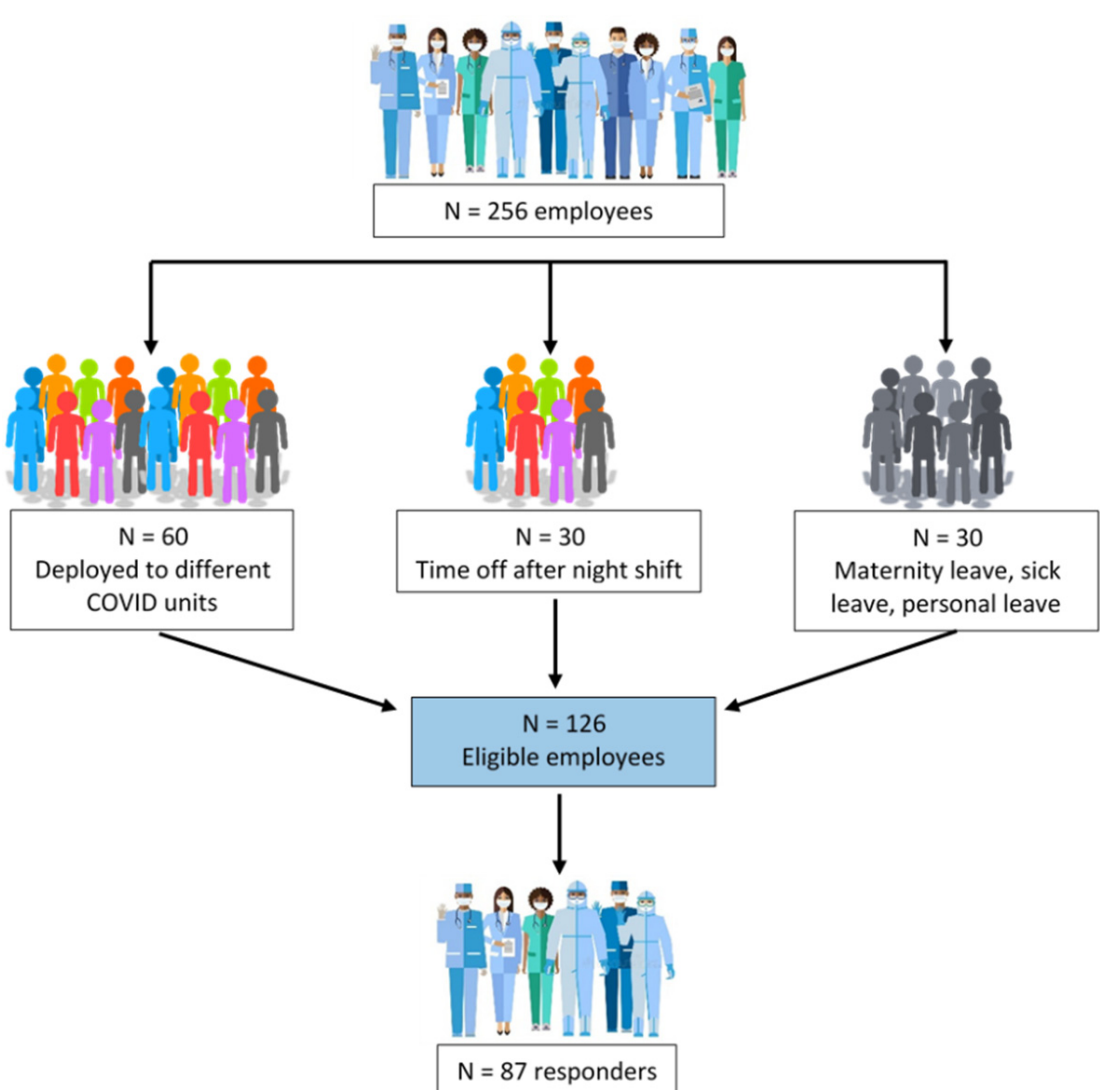
Enrollment flowchart.

Out of the 126 eligible participants, the study enrolled 87 adult employees of the ICU Clinic from the Emergency Clinical County Hospital in Târgu Mureș, Romania, with a response rate of 69% (Figure 1).

The ICU Clinic comprised medical, surgical, and trauma patients.

The study comprised three main groups of responders: doctors (D—*n* = 49), nurses (N—*n* = 27), and non-medical personnel, auxiliary (A—*n* = 11).

Out of the 87 persons who accepted to be part of the study, 73.5% (*n* = 64) were women and 26.5% (*n* = 23) were men. The groups of nurses and the auxiliary personnel were entirely formed by women.

The descriptive statistics of our data are shown in Table 1.

When the groups were compared between them related to age, working years, and COVID time criteria, we obtained a significant value (*p* < 0.05) between doctors and the rest of the groups regarding the age of the responders. The mean age of doctors was significantly lower than the rest of the groups. Another significant result was the time spent in the COVID unit. After group comparison, the auxiliary group spent significantly more days in the COVID unit than the participants from the other group results (*p* < 0.05).

The psychological test results and descriptive statistics of the three groups are presented in Table 2.

The anxiety test results obtained in each of the three groups were compared among the groups using the Kruskal—Wallis test. The statistical significance was not achieved in this regard, *p* > 0.05.

Spearman correlation of the data from the responders showed significant positive correlations between TPST and STAI tests (Table 3).

## 4. Discussion

This study’s results show that medical and auxiliary personnel from understaffed ICUs present with increased anxiety and post-traumatic stress.

COVID-19 was a biological threat that impacted the whole world in different aspects. In these past years, intensive care, which up until then was a medical specialty not so much on display, played a major role in the pandemics. The impact was rapid and harsh, and all the intensive care units had to adapt fast to the new situation [9].

Intensive care is a medical specialty that cares for patients in need of advanced medical support with medical personnel who is mentally prepared to face critical and deadly situations; however, during the pandemic, it turned out that, with an increased number of patients in a short time, the drama in every case, the impossibility to treat them properly because of a lack of oxygen or lack of beds or medication, had a supplementary effect upon ICU medical and non-medical personnel [8].

Our study results show that doctors spent more time in COVID-19 wards—when looking at the maximum number of days spent in COVID wards—than the questioned nurses and auxiliary personnel. This result can be explained by the shortage of ICU doctors in Romania [1]. Another reason is that during the pandemic, nurses and other personnel from non-ICU clinics could be more easily relocated to work in the COVID ICU. They could be trained quicker to help with the basics. The ICU specialists, on the other hand, could not be brought from other clinics on the same premises.

The statistically significant difference registered regarding the number of years worked in the field, the difference with a wide range in the doctor’s group—from 0.5 years to 41 years—ensues from the inclusion of the trainees in this group.

Post-traumatic stress disorder and burnout are known to be present among ICU personnel long before COVID-19 [12]. In our study, this disorder accounts for under ten percent of the doctor’s group. This result has a resemblance to the literature, although the study group is small and the responder’s honesty when answering the survey can be questioned [9,13,14]. Another explanation for these results only in the doctor’s group, as opposed to none and close to none in the other formed groups, is that doctors bear much more responsibility when it comes to patients, whereas nurses and non-medical personnel are mostly executors, not deciders in patient care.

Anxiety results from our study are moderate in all groups, much higher—more than half of the responders scored for moderate to high anxiety—than reported in other studies [15,16,17]. An explanation for the increased anxiety in all the groups could be the increased workload and the supplementary stress of COVID pandemics. A possible bias for these high percentages is the small number of responders per group, despite the response rate, which, according to the flowchart (see Figure 1) is almost 70%.

Our results showed that time spent working with COVID has a significant negative correlation with both age and the number of working years. This result can be explained by the fact that during the pandemic, the hospital hired new medium and auxiliary personnel for COVID units, and these were young nurses and young non-medical personnel.

Positive significant correlations were observed when the anxiety tests and post-traumatic tests were analyzed. This is somewhat expected because post-traumatic stress, which proved to be present among all the analyzed personnel categories, is directly correlated with both types of anxiety, state, and trait. These results have a good resemblance with the literature [17,18,19,20].

The COVID-19-related anxiety decreased due to habituation, disease understanding, the availability of protective equipment, and newly emerged treatments [21,22]. This aspect was noticed in our results—the correlation between time spent in COVID wards was inversely correlated with the number of working years.

COVID-time correlated, in all groups, significantly and negatively with age and working years. This result can be explained by the way hiring politics at the time, many young personnel were hired in the COVID unit throughout the country, and they spent many months in the COVID ICU. The same explanation applies to the significant and negative correlation between COVID time and the number of working years.

Our study showed a small picture of the psychological effects of COVID-19 upon the frontline medical workers. These evaluations are very important because, even before COVID, the ICU workers were exposed to burnout during work or when they were off work and were negatively influenced [23,24,25,26].

This study has limitations, such as the low number of participants, the voluntary nature of the survey could create a bias, and the participants are not to be considered representative for the evaluation. In this respect, the inclusion of more than one medical center is sought to reduce these biases.

## 5. Conclusions

All ICU personnel who dealt with COVID-19 patients present with moderate anxiety and post-traumatic stress disorder symptoms.

The doctors managed their COVID-19-related anxiety better than nurses and the auxiliary personnel because, although they spent more time in COVID units, they scored more for moderate anxiety than the other included groups, which spent less time in COVID units.

## Figures and Tables

**Table 1:** Descriptive statistics of age, working years, and COVID time for the studied groups. YRS-years Min—Minimum; Max—Maximum; SD—Standard Deviation; IQR—Inter Quartile Range; *—statistically significant.

	Doctors	Nurses	Auxiliary Personnel
	(*N* = 49)	(*N* = 27)	(*N* = 11)
Age (yrs)			
Min	25	22	36
Max	66	55	49
Mean ± SD	30.73 ± 7.72	40.44 ± 7.96	41.27 ± 4.07
Median	28	42	40
IQR	3	8	6
*p*-value	*	*	*
Working yrs			
Min	0.4	0.6	4
Max	41	35	32
Mean ± SD	5.29 ± 7.84	16.38 ± 8.95	17.36 ± 7.87
Median	3	16	19
IQR	3.25	10	7
*p*-value	*	*	*
COVID time (dys)			
Min	0	0	0
Max	153	122	122
Mean ± SD	34.94 ± 39.55	27.96 ± 39.43	63.55 ± 44.3
Median	21	3	61
IQR	58.5	61	94
*p*-value	*	*	*

**Table 2:** Descriptive statistics of the psychological tests. TPST—Post-Traumatic Stress Test; STAI-X1—State-Trait Anxiety Inventory—state; STAI-X2—State-Trait Anxiety Inventory—trait; Min—Minimum; Max—Maximum; SD—Standard Deviation; IQR—Inter Quartile Range.

	Doctors	Nurses	Auxiliary Personnel
	(*N* = 49)	(*N* = 27)	(*N* = 11)
PTST			
Min	0	0	0
Max	34	27	33
Mean ± SD	8 ± 7.89	7.18 ± 6.77	8.36 ± 10.39
Median	6	6	4
IQR	10	7	6
*p*-value	0.95	0.95	0.95
STAT-X1			
Min	20	20	22
Max	69	55	68
Mean ± SD	37.24 ± 9.79	35.7 ± 8.66	37.64 ± 12.48
Median	35	37	37
IQR	11.5	2	13
*p*-value	0.87	0.87	0.87
STAT-X2			
Min	20	21	25
Max	59	49	56
Mean ± SD	35.16 ± 7.67	34.74 ± 6.56	41 ± 8.56
Median	34	36	40
IQR	11	11	8
*p*-value	0.06	0.06	0.06

**Table 3:** Spearman correlation (p values and the correlation coefficient) of the data from the study’s responders. TPST—Post-Traumatic Stress Test, STAI-X1—State-Trait Anxiety Inventory—state, STAI-X2—State-Trait Anxiety Inventory—trait. *—statistically significant.

*p*	Age	Working Years	COVID Time	TPST	STAT-X1	STAT-X2
Age		0.00	*	0.99	0.87	0.85
Working years	0.00		*	0.55	0.74	0.44
COVID time	*	*		0.09	0.94	0.41
TPST	0.99	0.55	0.09		*	*
STAT-X1	0.87	0.74	0.94	*		*
STAT-X2	0.85	0.44	0.41	*	*	
**r**	**Age**	**Working Years**	**COVID Time**	**TPST**	**STAT-X1**	**STAT-X2**
Age		0.90	−0.28	0.00	−0.02	−0.02
Working years	0.90		−0.21	0.07	0.04	0.08
COVID time	−0.28	−0.21		−0.18	0.01	0.09
TPST	0.00	0.07	−0.18		0.65	0.57
STAT-X1	−0.02	0.04	0.01	0.65		0.59
STAT-X2	−0.02	0.08	0.09	0.57	0.59	

## Data Availability

The data used for this study can be found in the database of the Târgu Mureș Emergency Clinical County Hospital, Romania.

## Supplementary Materials

The following supporting information can be downloaded at: https://www.mdpi.com/article/10.3390/healthcare10071160/s1, Questionnaire S1: This study evaluates the stress level of medical and non-medical personnel during Covid-19 pandemic.
