# Supplementary material for: Survey on Anxiety and Post-Traumatic Stress Disorder in Intensive Care Personnel during the COVID-19 Pandemic in a Medically Under-Resourced Country
Source: Healthcare (Basel). 2022 Jun 22;10(7):1160. doi: 10.3390/healthcare10071160 (PMC9322630; doi:10.3390/healthcare10071160)
Supplement: Supplementary file 1 [file healthcare-10-01160-s001.zip › healthcare-1733593-supplementary.pdf]

This study evaluates the stress level of medical and non-medical personnel during Covid-19 pandemic

THE QUESTIONAIRES ARE ANONYMOUS

Age:

Sex:

Profession:

Professional degree:

Seniority level:

During Covid-19 pandemic you worked:

1) on Covid-19 Intensive Care ward
2) on non-Covid-19 Intensive Care ward
3) on both wards

If you worked on the Covid-19 Intensive care ward, please mention the **EXACT** period of working of the Covid-19 Intensive care ward:

1) ………………. days
2) ………………. weeks
3) ………………. months

A number of symptoms are presented below, especially experienced by a person after a traumatic event or events. Please tick on every row the intensity at which each symptom has affected you in the last month.

Symptom	Not at all	A little	Moderate	Very much
Annoying memories of the pandemic				
Nightmares of the pandemic				
The sensation of reliving the pandemic				
Anxiety or annoyance when seeing or hearing things related to the pandemic				
Avoiding things or situations that are reminiscent of the pandemic				
Lack of interest in professional or leisure activities				
Difficulties in feeling good or close to other people				
Emotional numbness				
Inability to imagine the future				
Sleeping difficulties				
Feeling of nervousness, irritability and anger				
Difficulty concentrating				
Inability to relax				

S.T.A.I. form - X1 –

INSTRUCTIONS: A number of statements which people have used to describe themselves are given below. Read each statement and select the appropriate response to indicate **<u>how you feel right now, that is, at this very moment</u>**. There are no right or wrong answers. Do not spend too much time on any one statement but give the answer which seems to describe your present feelings best.

Nr.	Description	Not at all	A little	Somewhat	Very much so
1.	I feel calm	1	2	3	4
2.	I feel secure	1	2	3	4
3.	I feel tense	1	2	3	4
4.	I feel strained	1	2	3	4
5.	I feel at ease	1	2	3	4
6.	I feel upset	1	2	3	4
7.	I am presently worrying over possible misfortunes	1	2	3	4
8.	I feel satisfied	1	2	3	4
9.	I feel frightened	1	2	3	4
10.	I feel uncomfortable	1	2	3	4
11.	I feel self-confident	1	2	3	4
12.	I feel nervous	1	2	3	4
13.	I feel jittery	1	2	3	4
14.	I feel indecisive	1	2	3	4
15.	I am relaxed	1	2	3	4
16.	I feel content	1	2	3	4
17.	I am worried	1	2	3	4
18.	I feel confused	1	2	3	4
19.	I feel steady	1	2	3	4
20.	I feel pleasant	1	2	3	4

S.T.A.I. form – X2 –

INSTRUCTIONS: A number of statement which people have used to describe themselves are given below. Read each statement and select the appropriate response to indicate **how you generally feel**. There are no right or wrong answers. Do not spend too much time on any one statement but give the answer which seems to describe how you generally feel.

Nr.	Description	Almost never	Sometimes	Often	Almost always
1.	I am in a good mood	1	2	3	4
2.	I get tired quickly	1	2	3	4
3.	I want to cry	1	2	3	4
4.	I wish I could be as happy as others seem to be	1	2	3	4
5.	I often miss some things because I cannot decide fast enough	1	2	3	4
6.	I feel rested	1	2	3	4
7.	I am "calm, cool and collected"	1	2	3	4
8.	I feel the difficulties piling up so that I cannot overcome them	1	2	3	4
9.	I worry too much over something that really does not matter	1	2	3	4
10.	I am happy	1	2	3	4
11.	I am inclined to take things too seriously	1	2	3	4
12.	I lack self-confidence	1	2	3	4
13.	I feel secure	1	2	3	4
14.	I try to avoid a critical moment or a difficulty	1	2	3	4
15.	I feel depressed	1	2	3	4
16.	I am content	1	2	3	4
17.	Some unimportant thought runs through my mind and bothers me	1	2	3	4
18.	I take disappointments so keenly that I cannot put them out of my mind	1	2	3	4
19.	I am a steady person	1	2	3	4
20.	I get in a state of tension or turmoil as I think over my recent concerns and interests	1	2	3	4
